# Attenuated Negative Feedback in Monocyte-Derived Macrophages From Persons Living With HIV: A Role for IKAROS

**DOI:** 10.3389/fimmu.2021.785905

**Published:** 2021-11-30

**Authors:** Celeste Faia, Karlie Plaisance-Bonstaff, Cecilia Vittori, Dorota Wyczechowska, Adam Lassak, Mary Meyaski-Schluter, Krzysztof Reiss, Francesca Peruzzi

**Affiliations:** ^1^ Department of Microbiology, Immunology, and Parasitology, Louisiana State University Health Sciences Center, New Orleans, LA, United States; ^2^ Stanley S. Scott Cancer Center, Louisiana State University Health Sciences Center, New Orleans, LA, United States; ^3^ Clinical and Translational Research Center, Louisiana State University Health Sciences Center, New Orleans, LA, United States; ^4^ Department of Interdisciplinary Oncology, Louisiana State University Health Sciences Center, New Orleans, LA, United States; ^5^ Department of Medicine and Department of Interdisciplinary Oncology, Louisiana State University Health Sciences Center, New Orleans, LA, United States

**Keywords:** HIV-1, Ikaros, monocytes, immune dysfunction, endotoxin tolerance

## Abstract

Persons living with HIV (PLWH) are at higher risk of developing secondary illnesses than their uninfected counterparts, suggestive of a dysfunctional immune system in these individuals. Upon exposure to pathogens, monocytes undergo epigenetic remodeling that results in either a trained or a tolerant phenotype, characterized by hyper-responsiveness or hypo-responsiveness to secondary stimuli, respectively. We utilized CD14^+^ monocytes from virally suppressed PLWH and healthy controls for *in vitro* analysis following polarization of these cells toward a pro-inflammatory monocyte-derived macrophage (MDM) phenotype. We found that in PLWH-derived MDMs, pro-inflammatory signals (*TNFA*, *IL6*, *IL1B*, miR-155-5p, and *IDO1*) dominate over negative feedback signals (*NCOR2*, *GSN*, *MSC*, *BIN1*, and miR-146a-5p), favoring an abnormally trained phenotype. The mechanism of this reduction in negative feedback involves the attenuated expression of IKZF1, a transcription factor required for *de novo* synthesis of RELA during LPS-induced inflammatory responses. Furthermore, restoring IKZF1 expression in PLWH-MDMs partially reinstated expression of negative regulators of inflammation and lowered the expression of pro-inflammatory cytokines. Overall, this mechanism may provide a link between dysfunctional immune responses and susceptibility to co-morbidities in PLWH with low or undetectable viral load.

## Introduction

Proper adherence to combined antiretroviral therapy (cART) can efficiently suppress viral replication and increase life expectancy of PLWH. However, even with prolonged HIV suppression, persistence of the virus in these individuals results in chronic inflammation and an increased risk of developing HIV-defining and non-HIV-defining illnesses, such as secondary infections, cardiovascular disorders, and cancer. Among the mechanisms contributing to chronic inflammation, microbial product translocation from the gut lumen to the blood leads to increased plasma levels of lipopolysaccharide (LPS) (1–8), which can persist even in virally suppressed PLWH. This suggests that LPS could chronically affect innate immune cell function.

Monocytes and macrophages are essential components of our innate immune system. Macrophages are present both in tissues as resident cells and can be recruited from the circulating pool of monocytes in the blood (9). Activation of macrophages involves dynamic regulation of epigenetic mechanisms that can be grossly divided into three categories: post-transcriptional histone modifications, DNA methylation, and changes in expression of non-coding RNAs (10). In non-activated monocytes/macrophages, histone repression marks (H3K9me3, H3K27me3, and H4K20me3) decorate chromatin at specific loci (11). After stimulation with Toll-like receptor (TLR) ligands such as LPS, repression marks are removed while activation marks (H3K4me1, H3K4me3, and H3K27ac) are deposited at promoters and enhancers (11). The balance of repression and activation marks present after an initial stimulus will determine the response to a secondary insult by establishing either a protective, short-term refractory functional state (*tolerance*), characterized by the inability of these cells to overproduce pro-inflammatory cytokines (12), or an elevated responsiveness (*trained immunity*), characterized by an increased production of pro-inflammatory cytokines in order to swiftly clear an infection (13–16). Importantly, an imbalance in the immune response can lead to long-term hyper-activation that underlies atherosclerosis and other inflammatory disorders (17, 18) or contributes to cancer (19).

Increasing evidence supports a key regulatory role for microRNAs (miRNAs) in the development, differentiation, and function of different types of immune cells, such as B cells, T cells, dendritic cells, and monocytes/macrophages (20–28). In particular, miR-146a-5p is a well-known anti-inflammatory miRNA involved in innate immunity (29–32) through inhibition of specific signaling molecules in the TLR4 pathway (33–37). Additionally, pro-inflammatory miR-155-5p is rapidly upregulated in myeloid cells during inflammation by a variety of TLRs and inflammatory cytokines (38), and its peripheral blood expression in HIV^+^ individuals correlates with T-cell activation and exhaustion (39). The coordinated regulation of both miR-146a-5p and miR-155-5p is critical for endotoxin-induced tolerance (40, 41).

IKZF1/IKAROS is a zinc-finger transcription factor associated with chromatin remodeling and is essential for normal hematopoiesis (42). IKAROS acts as a tumor suppressor (43) whose loss of function is associated with the malignant transformation of hematopoietic cells (44). While the critical role of IKAROS in T and B cell development and function is documented (44, 45), its role in monocytes and macrophages is largely unknown.

In this study, we tested the responsiveness of monocyte-derived macrophages (MDMs) obtained from PLWH to LPS stimulation and the establishment of endotoxin tolerance. Indeed, this phenotype was exhibited by control cells; however, PLWH-MDMs failed to establish endotoxin tolerance and instead showed a hyper-responsiveness suggestive of an abnormal development of a trained phenotype in this setting. Importantly, we have identified an attenuated miR-146a-5p- and IKZF1/RELA- mediated negative feedback as a mechanism likely involved in the increased production of cytokines in PLWH-MDMs.

## Results

### PLWH-MDMs Show Hyper-Responsiveness and Fail to Establish Endotoxin Tolerance

**Figure 1:**
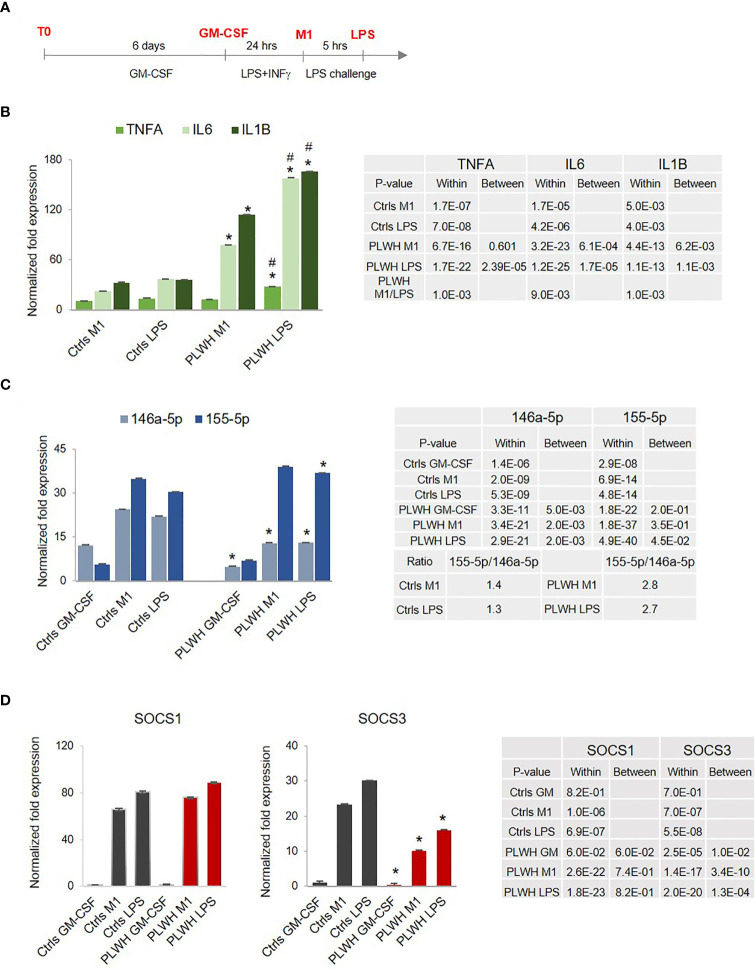
Hyper-responsiveness of PLWH-MDMs. **(A)** Diagram showing the experimental approach to mimic endotoxin tolerance in control- (Ctrls) and PLWH-MDMs (PLWH). In red are the four different experimental stages of monocyte cultures: T0, GM-CSF, M1, and LPS. **(B)** Normalized fold expression of *TNFA*, *IL6*, and *IL1B* mRNAs in MDMs over GM-CSF values. ^#^Indicates statistical significance between M1 and LPS in PLWH group. **(C)** Relative expression of miR-146a-5p and miR-155-5p in control- and PLWH-MDMs in the indicated treatments represented by the normalized fold change over T0. The ratio between miR-155-5p and miR-146a-5p in Ctrls and PLWH is shown in the Table **(D)** Relative expression of *SOCS1* and *SOCS3* mRNAs. In all graphs, error bars indicate SEM; *indicates statistical significance between groups (Ctrls, n=10, and PLWH, n=25) in the same cell treatments (M1 or LPS). The tables report specific *P*-values calculated within each group and between groups.

Endotoxin tolerance (ET) is a state of immune hypo-responsiveness that occurs as a result of prolonged stimulation with TLR agonists, including LPS, or other TLR-independent signals (46). When subjected to two consecutive stimulations with LPS, immune tolerant macrophages will produce less IL-1β, TNF-α, and IL-6 cytokines after the second treatment with LPS compared to the first one (47, 48). Therefore, we evaluated mRNA expression levels of these cytokines in circulating monocytes (CD14^+^/CD16^-^, T0) and MDMs obtained from controls (Ctrls,n=10) and from HIV^+^ individuals on cART (PLWH, n=25). Demographic information for the complete study cohort can be found in **Table 1**. CD14^+^/CD16^-^ cells were cultured for 6 days in the presence of GM-CSF (GM), and subjected to a low-dose treatment with LPS (M1) followed by a high-dose treatment with LPS (LPS), as described in methods and in **Figure 1A**. Total RNA was isolated and subjected to RT-qPCR assays according to our previously described protocols (49–53). Expression of *IL1B* and *TNFA* did not change significantly between PLWH and controls, while *IL6* was undetectable in most of the samples (both PLWH and controls) at T0 (data not shown). When normalized to their own GM-CSF treatment, higher levels of *IL1B* and *IL6* were already observed in M1-polarized (low-dose LPS) PLWH-MDMs compared to controls, and all three factors increased even further after the second, higher-dose of LPS (**Figure 1B**). Of note, the increased expression of all three cytokines in PLWH after the second dose of LPS was statistically significant compared to the M1 polarized state, with p-values of 0.001, 0.009, and 0.001, respectively (indicated with # in **Figure 1B** and in the table next to the graph). This hyper-responsiveness indicates a malfunction of PLWH-MDMs in establishing ET.

**Table 1:** General demographics of study cohort subjects.

Sample size	PLWH	Controls
	n = 95	n = 50
**Men**	61%^#^	40%
**Women**	39%^#^	60%
**Avg Age**	53*	46
**African- American**	88%	0%
**White**	22%^#^	99%
**High Viral Load (<400 copies/ml)**	23%	
**Low Viral Load (≤20 copies/ml)**	66%	
**CD4 T cell count**	532 cells/mm^3^ (range 90 to 1233)	
**cART**	100%	0%
**Smoking**	53%	0%
**Hypertension**	42%	0%
**Diabetes**	27%	0%

Subsets of PLWH and controls from this larger cohort were indiscriminately utilized throughout various experiments. ^#^χ^2^ indicates statistical significant difference in gender distribution within the group of PLWH and between PLWH and Controls; *Student’s t-test indicates statistical significance difference between PLWH and Controls.

We further evaluated expression levels of two miRNAs known to play a role in inflammation and endotoxin tolerance (32, 41, 48, 54), the anti-inflammatory miR-146a-5p and the pro-inflammatory miR-155-5p. The relative expression of these two miRNAs at T0 (freshly isolated monocytes) showed no statistically significant differences between Ctrls and PLWH (data not shown). Changes became apparent when the cells were exposed to LPS. Results in **Figure 1C** show significantly lower expression of miR-146a-5p in PLWH-MDMs compared to controls, while miR-155-5p expression was consistently higher in PLWH-MDMs. These data indicated co-regulated expression of miR-155-5p and miR-146a-5p in control MDMs after LPS induction, corroborating a previous report (41). However, coordinated expression of these two miRNAs in the PLWH-MDMs was lost. This loss significantly affected the miR155/miR146 ratio, which in PLWH-MDMs was 2 fold higher than in controls, as indicated in the table (**Figure 1C**).

Directly associated with the TLR4-induced cytokine production is the expression of the suppressor of cytokine signaling (SOCS) family of proteins, whose upregulation is secondary to LPS exposure as part of the negative feedback loop that normally counteracts an inflammatory response (55). We evaluated the expression of *SOCS1* and *SOCS3* throughout the four stages (T0, GM-CSF, M1, and LPS challenge) and found that *SOCS1* mRNA was highly but almost equally induced in both control- and PLWH-MDMs at both M1 and LPS challenge conditions, while *SOCS3* expression was significantly lower in PLWH-MDMs compared to controls in the same experimental conditions (**Figure 1D**). Altogether, these data indicate a defect of PLWH-MDMs in building a negative feedback loop that would counteract the inflammatory response.

### ChIP-Sequencing Reveals Substantial Differences Between HIV^+^- and Control-Derived Monocytes That Were Magnified by *Ex Vivo* Functional Assays

**Figure 2:**
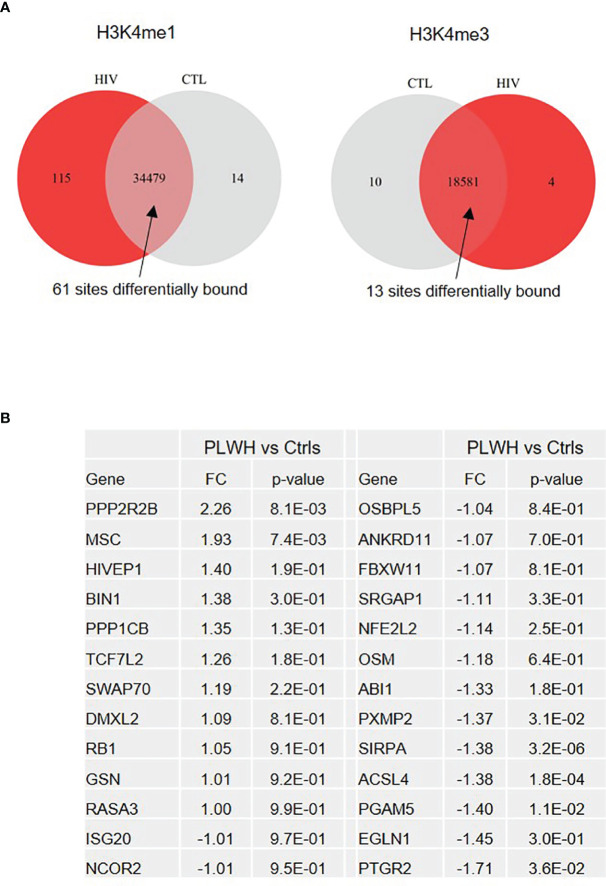
ChIP-sequencing reveals genes differentially expressed in freshly isolated monocytes from PLWH compared to controls. **(A)** Venn diagram showing the number of sites enriched in H3K4me1 and H3K4me3 marks uniquely found in PLWH and controls, as well as those at sites common in both sets of samples (Ctrl n=3; PLWH n=6). Note that the unique sites in one group means that they were present in all the samples from that group and absent in all samples of the other. A false discovery rate (FDR) < 0.05 was applied. **(B)** List of 26 differentially expressed genes in freshly isolated monocytes from PLWH (n=25). Relative quantification was calculated over controls (Ctrls, n=10) T0. For the complete list of genes, see the Excel file provided in **Supplementary Material**.

Freshly isolated CD14^+^ monocytes obtained from 3 control subjects (Ctrls) and 6 PLWH were fixed and subjected to ChIP-sequencing (Diagenode Inc, NJ). ChIP was performed using H3K4me1 (enriched at enhancers) and H3K4me3 (enriched at promoters) antibodies, and DNA from 18 ChIPs (9 samples x 2 Abs) was sequenced. The Venn diagram in **Figure 2A** displays the number of chromosome sites enriched in H3K4me1 and H3K4me3 that were uniquely found in HIV-derived cells (115 and 4, respectively) or control cells (14 and 10, respectively). Of the sites common to both HIV-derived cells and controls, 61 and 13 were found to be differentially enriched in HIV^+^ subjects in H3K4me1 and H3K4me3, respectively. Please see the Excel file **Supplemental Material** for the summary of ChIP-sequencing data.

**Figure 3:**
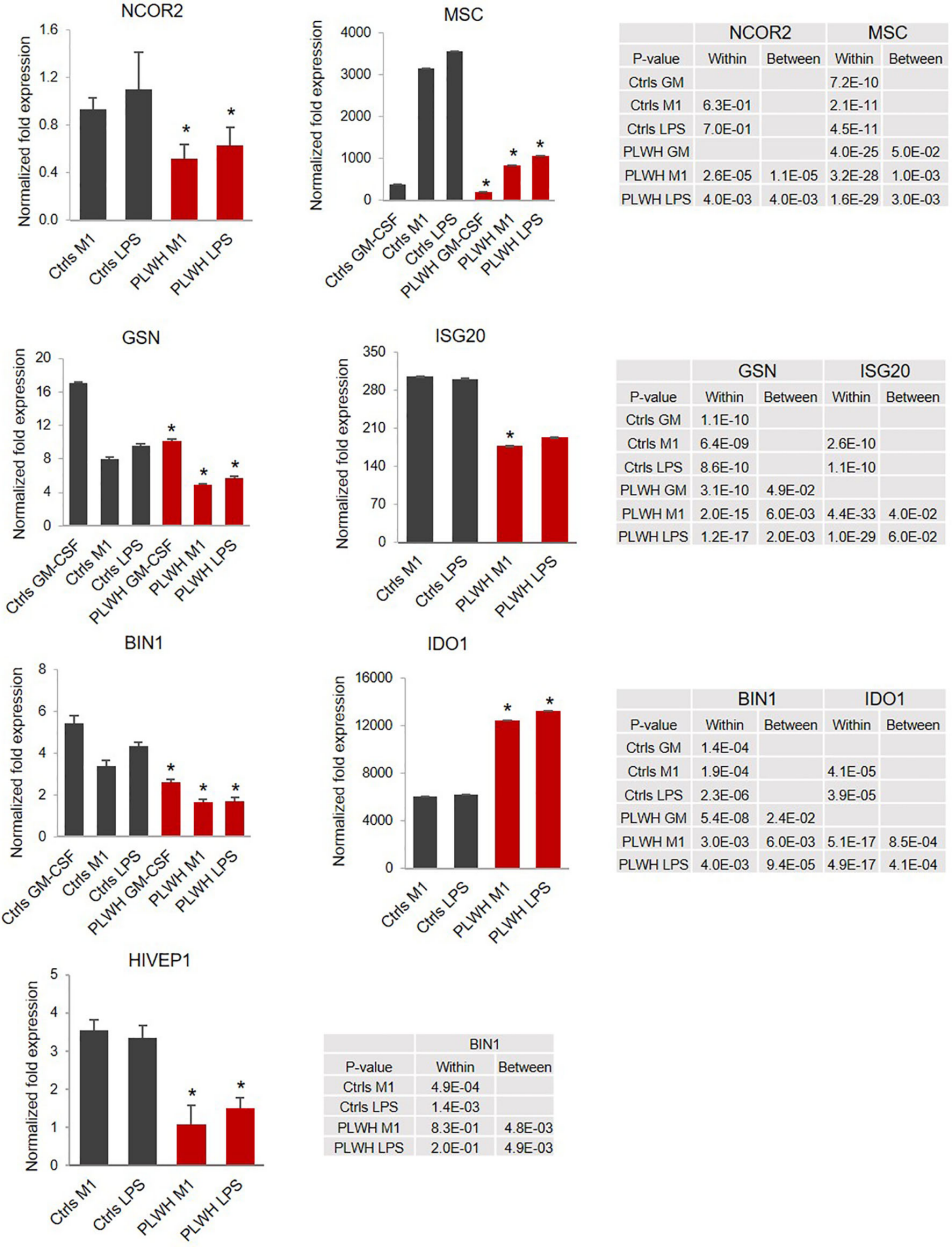
Relative expression of selected genes indicates impaired upregulation of negative regulators of LPS-triggered signaling. Relative expression of the selected mRNAs was calculated over T0 (*GSN*, *MSC*, and *BIN1*) or GM-CSF (*NCOR2*, *ISG20*, *IDO1*, and *HIVEP1*). The graphs represent the normalized fold change. Error bars indicate SEM; *indicates statistically significance between groups (Ctrls, n=10; PLWH, n=25). The tables report specific *P*-values calculated within each group and between groups.

Of the unique and common sites, we selected 26 genes involved in inflammation and transcriptional regulation for further analysis using monocytes from 25 PLWH and 10 age- and gender-matched HIV- controls. **Figure 2B** shows relative expression (fold change, FC) of the 26 selected genes in freshly isolated monocytes (T0) of PLWH compared to controls. Next, we sought to investigate whether expression of these genes would change in monocytes from PLWH and controls during the inflammatory response. For each sample, cells were cultured following the experimental set up described in **Figure 1A**. Confirming the ChIP-seq data, *NCOR2*, *GSN*, *MSC*, and *BIN1* were found to be differentially regulated in PLWH-MDMs and control-MDMs (**Figure 3**). These four genes are negative regulators of transcription and/or negative regulators of inflammation. **Figure 3** shows the relative expression of these genes compared to T0 or GM-CSF. NCOR2 (nuclear receptor corepressor 2) is a transcriptional repressor (56, 57) involved in anti-inflammatory pathways (58, 59). Musculin (MSC) is a basic helix-loop-helix (bHLH) transcription factor (TF) that inhibits other bHLH TFs. Gelsolin (GSN) is an actin-binding protein that inhibits the inflammatory process induced by LPS (60). Interestingly, another IFN-induced gene, *ISG20*, was less expressed in PLWH-MDMs compared to controls. The anti-viral factor ISG20 inhibits translation of ectopically introduced genetic material, for instance through viral infections (61).

The bridging integrator 1 (BIN1) is an adaptor protein with potential anti-inflammatory function (62) promoted *via* negative regulation of indoleamine 2,3-dioxygenase (IDO1), which is the first and rate-limiting enzyme in the kynurenine pathway of tryptophan metabolism (63). Indeed, in addition to lower expression of *BIN1* (**Figure 3**), we found a significant upregulation of *IDO1* in HIV-derived cells, further supporting repression of this anti-inflammatory loop in PLWH-derived MDMs. HIVEP1 is a zinc-finger protein that binds specific DNA sequences present in the enhancers of viral and cellular promoters (64, 65). We found expression of *HIVEP1* mRNA significantly lower in PLWH-MDMs compared to controls (**Figure 3**).

### Attenuation of the IKZF1/RELA Pathway in PLWH-MDMs

**Figure 4:**
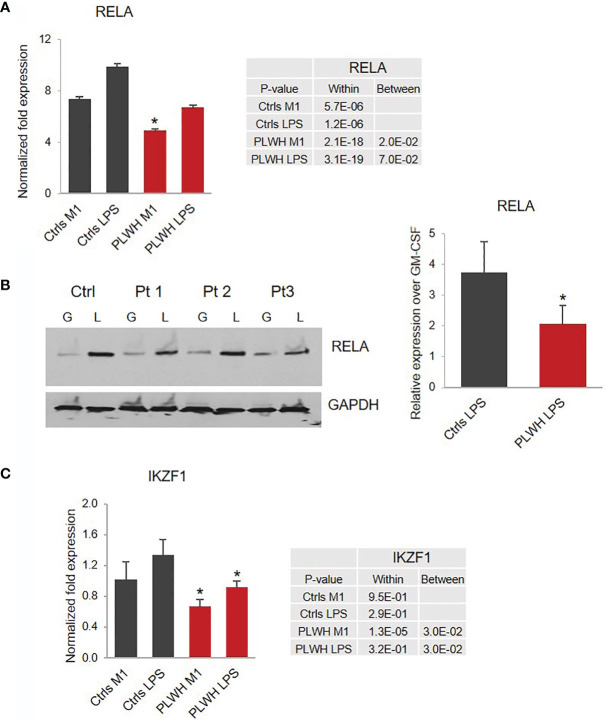
Impaired expression of RELA and IKZF1 in PLWH-MDMs compared to controls. **(A)** Relative expression of *RELA* mRNA calculated over T0. Error bars indicate SEM; *indicates statistical significance between groups (Ctrls, n=10; PLWH, n=25). **(B)** Expression of RELA protein in MDMs obtained from Ctrls (n=4) and PLWH (n=9) treated with GM-CSF (G) and LPS (L). Blots were quantified using image J software. The graph represents the ratio of RELA in LPS compared to GM-CSF after normalization with GAPDH (right panel). *Indicates statistical significance between Ctrls and PLWH. **(C)** Relative expression of *IKZF1* in controls (n=10) and PLWH (n=25). The tables report specific *P*-values calculated within each group and between groups.

Since NF-кB transcriptional complex plays a key role in LPS-induced inflammatory cytokine production, we sought to investigate the expression of the transcription factor RELA/p65 and NFKB1/p50, the most abundant subunits of the NF-кB complex. While we did not observe significant changes in the expression of *NFKB1* in PLWH-MDMs compared to controls (data not shown), there was a major difference in the induction of *RELA* expression in M1 and LPS in PLWH-MDMs (n=25) compared to controls (n=10) (**Figure 4A**). The mRNA data was confirmed by Western blot analysis in 4 Ctrl-MDMs and 9 PLWH-MDMs samples in the same experimental setting. **Figure 4B** shows significantly lower levels (*p*= 0.04) of RELA protein in PLWH-MDMs after LPS exposure compared to controls. These data could indicate a reduced transcription of *RELA* during M1 stimulation (IFN-γ + LPS).

**Figure 5:**
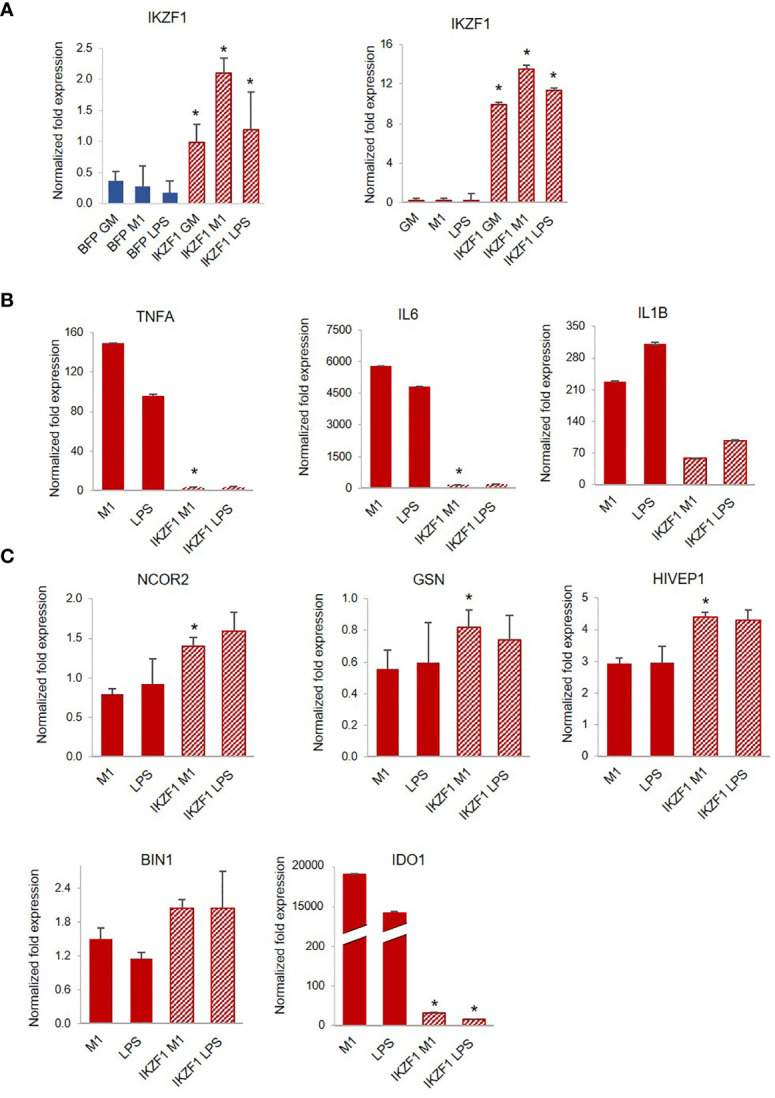
Transfection of IKZF1 into PLWH-MDMs reduces their hyper-responsiveness to LPS and partially restores the expression of negative regulators of inflammation. **(A)** Relative expression of *IKZF1* mRNA in IKZF1-transfected PLWH-MDMs (left panel, n=4) at day 3 or day 5 post-isolation (right panel, n=4) in the indicated treatments calculated over T0. BFP control vector or untransfected cells were used as negative controls at day 3 or day 5, respectively. Expression levels of pro-inflammatory cytokines *TNFA*, *IL6*, and *IL1B* **(B)** and negative regulators of inflammation *NCOR2*, *GSN*, *HIVEP1*, *BIN1*, and *IDO1* **(C)** in cells transfected at day 5 post-isolation (See **Figure S1** for data obtained after transfection at day 3). In all graphs, error bars indicate SEM; *indicates statistical significance between groups.

Among the molecules possibly implicated in the *de novo* transcription of *RELA* during exposure of macrophages to LPS is the zinc-finger protein IKAROS (66, 67). IKAROS is required for the new synthesis of *RELA* following LPS stimulation, and we found expression of *IKZF1*, the gene encoding IKAROS, significantly decreased in PLWH-MDMs compared to controls (**Figure 4C**). Importantly, *in silico* analysis of ChIP-seq data (68) indicate that most of the genes investigated in this project (*MSC*, *NCOR2*, *ISG20*, *RELA*, miR-146a-5p, and miR-155-5p) are also the transcriptional targets of both RELA and IKAROS (ENCODE transcription factor targets database). Altogether, this experimental evidence prompted us to hypothesize that the IKAROS/RELA axis is a key transcriptional mechanism that has lost function in PLWH-MDMs. To further investigate the role of IKAROS in the attenuated anti-inflammatory response, PLWH-MDMs (n=4) were transfected with a plasmid encoding IKZF1 on day 3 or 5 post-isolation, using a BFP empty vector or untransfected cells as negative controls, respectively. Cells were then treated as described in **Figure 1A** (GM-CSF, M1, and LPS). As expected, after each treatment, more *IKZF1* mRNA was present in transfected cells compared to BFP-transfected cells (**Figure 5A**, left panel) or untransfected cells (**Figure 5A**, right panel, and **Figure S1**), although the efficiency of transfection was higher and more consistent at day 5. We then investigated the response of IKZF1-transfected cells (day 5) to LPS treatments by measuring levels of pro-inflammatory cytokines and negative regulators of inflammation. We found that the presence of IKZF1 greatly attenuated expression of *TNFA*, *IL6*, and *IL1B* (**Figure 5B**), while increasing the expression of *NCOR2*, *GSN*, *HIVEP1*, and *BIN1* (**Figure 5C**), therefore corroborating our hypothesis that IKZF1/RELA plays a role in the hyper-responsiveness of PLWH-MDMs. As we previously observed (**Figure 3**), the upregulation of *BIN1* continued to correlate with downregulation of *IDO1* (**Figure 5C**). Specific p-values corresponding to **Figure 5** can be found in **Table S1**. Comparable data were obtained when IKZF1 was transfected at day 3 (**Figure S1**).

**Figure 6:**
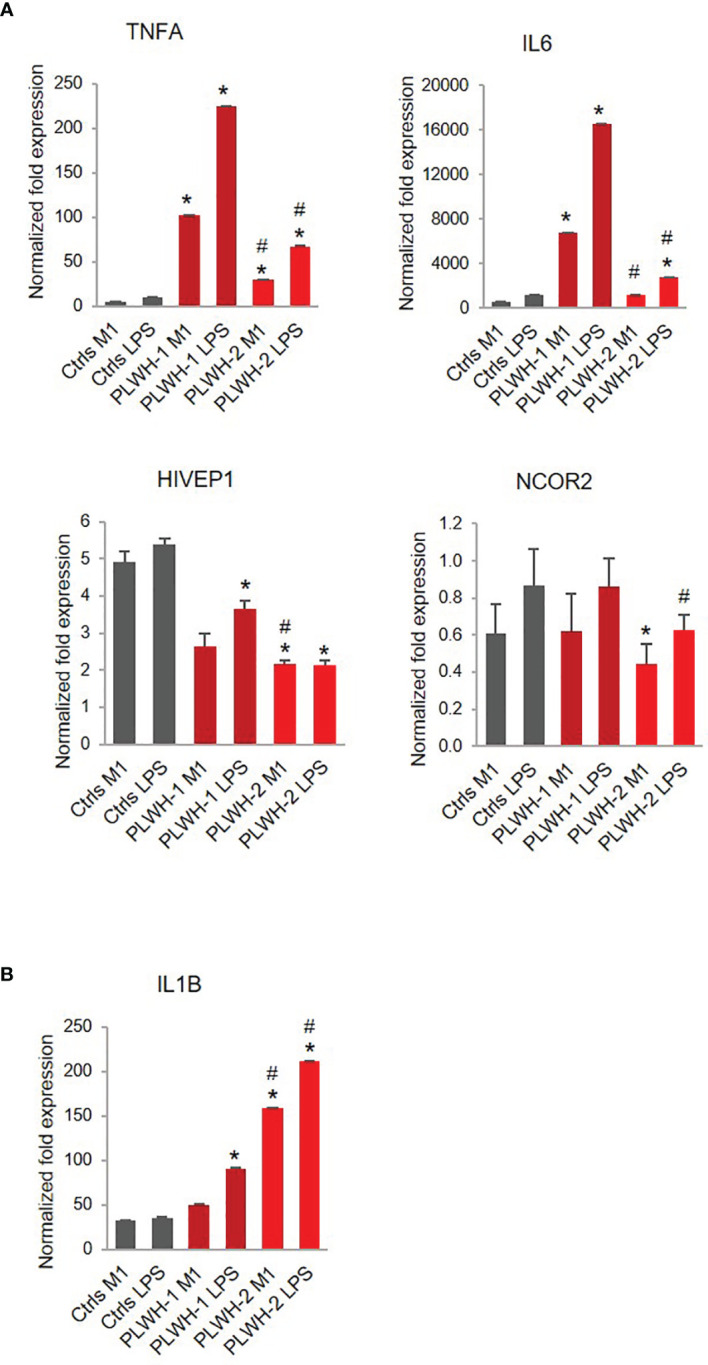
Stratification of PLWH-derived samples based on TNFA and IL6 expression levels. Bar graphs indicate mRNA expression levels of *TNFA*, *IL6*, *HIVEP1*, *NCOR2* **(A)** and *IL1B* **(B)** in controls (n=10), PLWH subgroup 1 (PLWH-1, n=7), and PLWH subgroup 2 (PLWH-2, n=18). *Indicates statistical significance between PLWH (1 or 2) and controls, while ^#^indicates statistical significance between PLWH-1 and PLWH-2.

Finally, while on average we found a hyper-responsive phenotype in PLWH-derived cells, unsupervised cluster analysis based on the mRNA expression levels of the pro-inflammatory factors *TNFA* and *IL1B* in M1-polarized cells determined two distinct subgroups of PLWH samples (**Figure S2**). Since the stronger effect of LPS on *TNFA* and *IL1B* levels was observed after the first (low-dose) treatment with LPS (M1), we chose those values for the cluster analysis. One subgroup showed drastic hyper-responsiveness to LPS (PLWH-1, n=7), while the other subgroup showed more moderate hyper-responsiveness (PLWH-2, n=18) (**Figure 6A**). Interestingly, expression of *HIVEP1* was significantly lower in both subgroups compared to controls, and expression levels of *NCOR2* were significantly lower only in subgroup 2 compared to controls and subgroup 1 (**Figure 6A**). In contrast to *TNFA* and *IL6*, expression of *IL1B*, while significantly greater in PLWH subgroup 1 LPS condition compared to controls, was further increased in PLWH subgroup 2 (**Figure 6B**). These results further support the notion that PLWH overall present a defective immune response because despite variation in gene expression among PLWH subgroups, pro-inflammatory factors and signaling pathway mediators generally remain significantly dysregulated compared to controls.

## Discussion

Despite low viral load and normal CD4^+^ T cell count, PLWH remain at a high risk of developing secondary illnesses, suggesting a dysfunctional immune system. Given the importance of innate immune cells in the inflammatory response, we sought to determine if monocytes from PLWH on combined antiretroviral therapy (cART) had functional differences compared to uninfected control cells when stimulated with LPS, following their polarization toward a pro-inflammatory M1-like macrophage phenotype. Specifically, we evaluated the ability of the cells to establish endotoxin tolerance measured by the expression of the pro-inflammatory cytokines *TNFA*, *IL1B*, and *IL6*, as well as two microRNAs, miR-146a-5p and miR-155-5p, which are key regulators of the inflammatory response (29–32, 48). We found increased expression of *TNFA*, *IL1B*, and *IL6* mRNAs in PLWH-MDMs compared to controls (**Figure 1B**), confirming results from recent studies that evaluated the function and phenotype of PBMCs (8) and MDMs (69) in PLWH. Importantly, the three pro-inflammatory cytokines showed higher expression in M1-polarized PLWH-MDMs compared to controls, indicating a trained phenotype.

We additionally found lower levels of miR-146a-5p and increased levels of miR-155-5p following LPS stimulation in PLWH-MDMs compared to controls (**Figure 1C**). This could be highly relevant considering that miR-155 mediates pro-inflammatory and miR-146, anti-inflammatory responses (27, 41, 70). Sustained levels of miR-155-5p correlate with increased cytokine expression and loss of endotoxin tolerance through targeting of SHIP-1, SOCS1, and BCL6 (71). Interestingly, a coordinated regulation of miR-155-5p and miR-146a-5p occurs in endotoxin tolerance and results in the mono-allelic expression of each miRNA (41). However, our data show a more than two-fold increase in miR-155-5p expression compared to miR-146a-5p in PLWH-MDMs (**Figure 1C**), perhaps indicating a loss of coordinated regulation of these two miRNAs. We further hypothesized that the hyper-responsiveness of PLWH-MDMs following consecutive exposures to LPS was due to epigenetic changes present in circulating monocytes of PLWH. To gain insights into potential epigenetic differences between freshly isolated CD14^+^ cells obtained from PLWH (n=6) and from HIV^-^ controls (n=3), we determined the presence of H3K4me1 and H3K4me3 enriched sites in these samples by ChIP-sequencing analysis (**Figure 2**). Results indicated important differences in these epigenetic marks in a set of genes involved in the negative regulation of inflammation (i.e. *NCOR2*, *GSN*, *MSC*, *ISG20*, *BIN1*, and *HIVEP1*). While these two markers of activation do not give an entirely comprehensive view of the mechanisms involved in gene expression –and may not directly indicate gene expression trends following immune stimulation- they provided a starting point from which we could evaluate specific pathways we may have otherwise overlooked. When we measured expression levels of those genes in MDMs grown in the presence of LPS, we found significantly lower expression of these genes in cells derived from PLWH compared to controls (**Figures 3**, **6**), further suggesting that the hyper-responsiveness of PLWH-MDMs could be due to attenuated expression of negative modulators of inflammation. NCOR2 mediates transcriptional silencing by promoting chromatin condensation, thus preventing access to basal transcription (56, 57). Like other members of the nuclear receptor superfamily of ligand-dependent transcription factors, NCOR2 can antagonize pro-inflammatory programs by altering the recruitment of co-activators and co-repressors (58, 59). Interestingly, *NCOR2* was identified as one of the host genes necessary for HIV infection, also called HIV-dependency factors (72), and two SNPs on the *NCOR2* gene were found to be associated with HIV transmission in a high throughput genome-wide analysis (73). GSN has been shown to inhibit inflammatory processes induced by LPS (60). Furthermore, increased levels of plasma gelsolin (pGSN) in untreated HIV-infection has been associated with disease severity (74). *HIVEP1*, which we found less expressed in PLWH-MDMs (**Figure 3**), plays a role in activating HIV-1 gene expression (65), as well as in both the classical and alternative polarization of macrophages (75). While NCOR2, GSN, and HIVEP1 seem to be associated with early HIV infection, our CD14^+^ monocytes are not infected with HIV, as determined by qPCR performed on both RNA and DNA extracted from these cells at T0 and after 7 days in culture (data not shown). Nevertheless, our data showing attenuated expression of *NCOR2*, *GSN*, and *HIVEP1* in cells from PLWH further support the established role of these factors as negative modulators of inflammation. BIN1 is involved in macrophage phagocytosis and possesses anti-inflammatory function (62) through the negative regulation of IDO1 (63). Consequently, in parallel to a reduced expression of *BIN1*, we found upregulation of *IDO1* in PLWH-derived cells (**Figure 3**). Interestingly, IDO1 protein was found to be elevated in the plasma of virally-suppressed PLWH (76–79). Given the importance of IDO1 in T cell function and HIV disease progression (77), our findings may provide a mechanism for the presence of IDO1 in the plasma of PLWH. In addition to transcriptional regulators, the interferon-induced factor *ISG20* showed reduced expression in PLWH-MDMs compared to controls (**Figure 3**). Altogether, our data indicated attenuation of negative feedback regulators of inflammation in monocytes/macrophages of PLWH. We then performed *in silico* analysis using IKZF1 ChIP-sequencing datasets from the ENCODE transcription factor database and found that *MSC*, *NCOR2*, *ISG20*, *RELA*, *HIVEP1*, *MIR146A*, and *MIR155* were gene targets of IKZF1. Previous literature indicates that IKAROS plays a dual role in orchestrating the inflammatory response in that it represses a subset of genes while activating others, as determined by transcriptomic and epigenetic analyses in primary macrophages exposed to LPS at various times (66). Furthermore, live single-cell imaging revealed a positive feedback loop involving the induction of RELA expression, which rewired the NF-кB regulatory network when cells were stimulated with LPS above a specific concentration (67). This reprogramming required IKAROS and indicated that RELA positive feedback can overcome existing negative feedback loops and enable cells to discriminate between different concentrations of LPS (67). Our data showing reduced expression of *IKZF1* and *RELA* in PLWH-MDMs (**Figure 4**) clearly indicate a dysfunctional IKAROS/RELA axis with impaired negative feedback loops that result in an abnormal pro-inflammatory response. Furthermore, overexpression of IKZF1 in PLWH-derived cells partially counteracted this imbalanced immune response (**Figure 5**). This novel mechanism could explain, at least in part, the persistent immune activation in PLWH and their increased risk of developing co-morbidities. Finally, stratification of PLWH-derived samples based on *TNFA* and *IL1B* cytokine expression revealed differences in mRNA levels of inflammatory mediators between two PLWH subgroups; however, these subgroups maintained overall dysregulation compared to controls (**Figure 6**). While microbial dysbiosis often observed in PLWH may explain the hyper-responsiveness to LPS treatments in *in vitro* functional assays, epigenetic changes and attenuation of negative feedback observed in this study suggest a more complex mechanism likely involving multiple factors, including age and cART among many others. Related to this, it should be noted that the HIV^-^ controls used in our study had none of the conditions reported for PLWH, such as hypertension and diabetes. Despite the sample size being large enough for statistical analysis, it was too small to consider variables other than the HIV infection status. Further studies are needed to determine the role of co-morbidities in the phenotype and function of monocytes. It is also of interest to this laboratory to expand the HIV^-^ control population in our studies to include African-American individuals.

**Figure 7:**
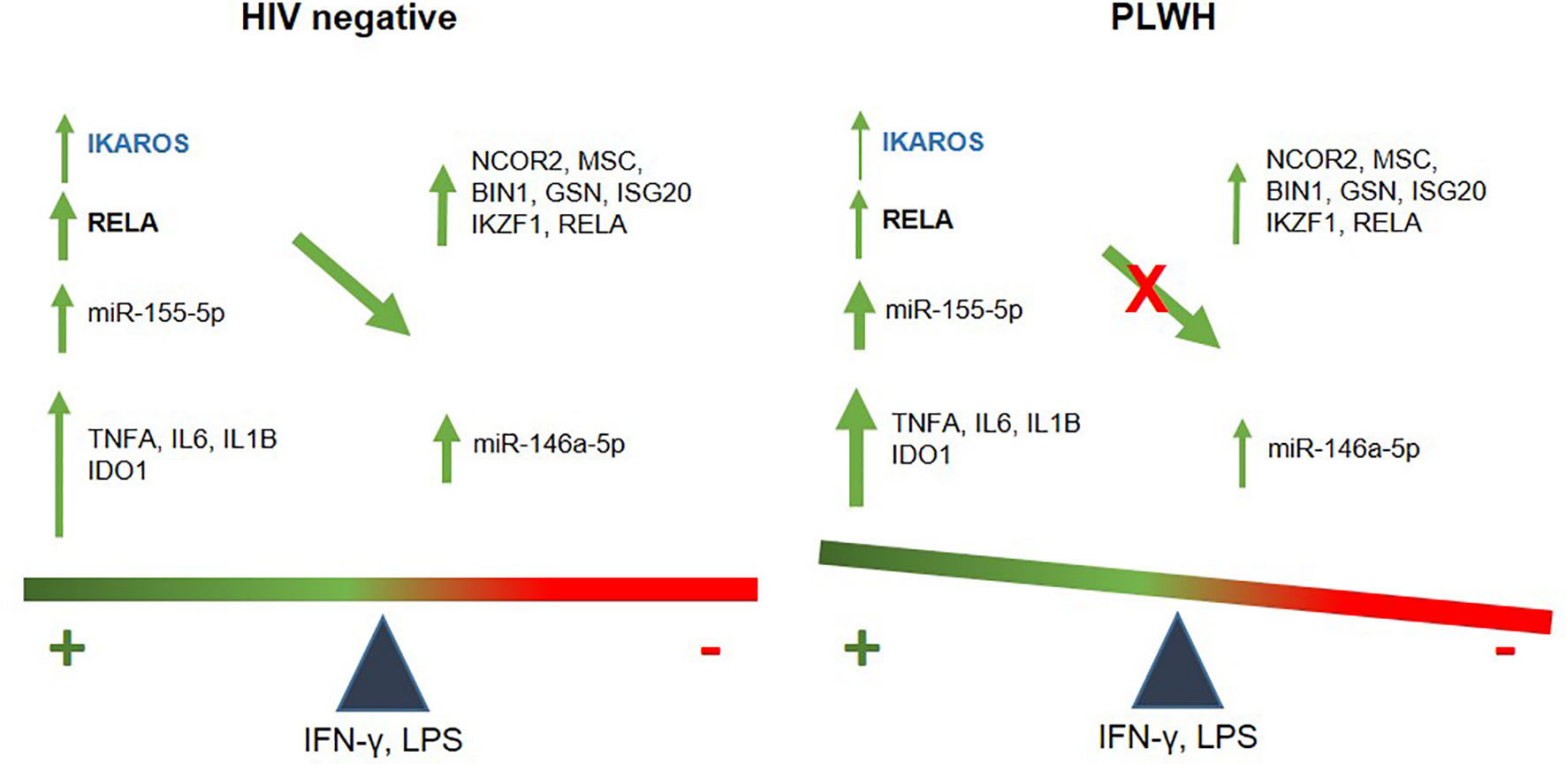
Schematic illustration of an imbalanced immune response in PLWH. In healthy controls, temporally-regulated positive (miR-155-5p, IDO1, IL6, TNFA and IL1B) and negative feedbacks (146a-5p, NCOR2, GSN, MSC, IKZF1, BIN1, ISG20, and RELA) will lead to a balanced immune response, with IKZF1 and RELA playing a central role in the transcription of negative regulators. Instead, attenuated IKAROS/RELA axis in PLWH-MDMs results in positive signals dominating over negative feedback. This imbalanced immune response may contribute to chronic inflammation and increase the risk of co-morbidities.

In summary, this study reveals a novel pathway of inflammatory dysregulation in PLWH that may have clinical implications in the treatment of ongoing co-morbidities in this population despite systemic control of HIV through cART. The pathway involves a dysfunctional IKAROS/RELA axis that is at least partially responsible for an attenuated anti-inflammatory response (**Figure 7**).

## Methods

### Sample Cohort

Blood samples were obtained from newly enrolled volunteers recruited in the ongoing collections of samples by the HIV-Clinical Tumor Biorepository (HCTB) core facility. Demographic information of PLWH and HIV^-^ controls collected for this study is presented in **Table 1**.

### Primary Cells

CD14^+/^CD16^-^ cells were isolated by negative selection (EasySep™ Direct Human Monocyte Isolation Kit, STEMCELL Technologies, Vancouver, BC, Canada) from 40 ml of whole blood from volunteers (PLWH and HIV^-^ controls) and plated for M1 polarization and LPS stimulation. Cells were cultured for 6 days in RPMI/10% FBS/GM-CSF [25 ng/ml] then given LPS [100 ng/ml, Sigma Aldrich, St. Louis, MO] and IFNγ [20 ng/ml] in 5% FBS for 24 h, as we and others have reported (80, 81). Hyclone Defined FBS was from Gibco (New York, NY). Human GM-CSF and IFN-γ were from R&D Systems (Minneapolis, MN). To mimic endotoxin tolerance, cells were exposed to a higher concentration of LPS [1 µg/ml; LPS challenge] for an additional 5 hours. Cell purity and macrophage polarization (development to M1) were assessed with flow cytometry, as we previously reported (81).

### RT-qPCR and Primers

Primers to amplify miR-146a-5p and miR-155-5p, as well as primers specific to GAPDH, 5S ribosomal RNA, and IDO1 were purchased from Qiagen (Germantown, MD). All other primers were from IDT (Coralville, IA) and are listed in **Table S2**. RNA was extracted using miRNeasy Mini kit (Qiagen). MiRCURY LNA RT and RT2 First Strand kits (Qiagen) were used to generate cDNA for miRNA or gene expression analysis, respectively. SYBRGreen mix was from Qiagen. Quantitative real time PCR was performed using a Roche LightCycler 480 instrument. PCR conditions were 10’ at 95°C for 1 cycle and then 15” at 95°C and 1’ at 60°C for 45 cycles. PCR data for mRNAs and miRNAs were normalized using GAPDH and 5S ribosomal RNA, respectively. Relative quantification was calculated according to the formula 2^-ΔΔCt^. Data were subsequently converted to log- normalized fold change, and the standard error was calculated following formulas described in (82). Two tailed t-test was applied to determine statistically significant differences in miRNAs and gene expression within each group (e.g. M1 or LPS *vs* GM-CSF in the Ctrl group), as well as between the groups [controls (Ctrls) *versus* PLWH]. Most of the graphs show qPCR data compared to the GM-CSF treatment; however, when we observed statistically significant differences related to the GM-CSF treatment, relative quantification was performed using T0 (**Figures 1**, **3**).

### Antibodies

Cells were lysed in modified RIPA buffer containing protease and phosphatase inhibitors. Protein lysates (25-30 µg) were subjected to standard Western blotting using SDS-PAGE and probed for total RELA (NF-κB p65) (Cell Signaling Technology, Danvers, MA). GAPDH (Santa Cruz Biotechnology, Dallas, TX) was used as loading control.

### Transfection

PLWH-derived MDM cells cultured for 5 days were transfected with 3 µg IKZF1 plasmid (pLV[Exp]-EGFP:T2A:Puro-CMV>hIKZF1[NM_006060.5, VectorBuilder, Chicago, IL) or the empty vector BFP (pLenti6.2-mTagBFP2, Addgene #113725) using the ViaFect transfection reagent (Promega; Madison, WI).

### ChIP-Sequencing

In accordance with Diagenode (Denville, NJ) recommendations for fixation and preparation of cells for ChIP-Seq, 8x10^6^ primary monocytes isolated at T0 were pelleted and resuspended in PBS containing formaldehyde (37%) followed by the addition of 1.25 M Glycine. Cells were washed twice with cold PBS and 2x10^6^ aliquots were shipped to Diagenode for subsequent ChIP-Seq.

### Statistics

RT-qPCR relative expression data was analyzed using the method described in (82). *P* values were calculated using two-tailed student t-test.

### Study Approval

Blood samples were obtained from volunteers recruited in the HIV-Clinical Tumor Biorepository (HCTB) core facility, following protocols described in our previous studies (50, 53, 81). The process for establishing a detailed operational protocol for the collection, de-identification, transportation, and storage of these samples has been approved by the LSUHSC-NO Institutional Review Board (IRB). Demographic information, viral load, CD4^+^ T cell counts, co-morbidities, and adherence data are available through the HCTB.

## Data Availability Statement

The datasets presented in this study can be found in online repositories. The names of the repository/repositories and accession number(s) can be found below: https://www.ncbi.nlm.nih.gov/, accession ID: GSE185405.

## Ethics Statement

The studies involving human participants were reviewed and approved by Louisiana State University Health Sciences Center New Orleans. The patients/participants provided their written informed consent to participate in this study.

## Author Contributions

FP conceived the study. CF performed all the experiments. KP-B and MM-S provided clinical samples. AL contributed to plasmid preparation. CV and DW helped with RNA extraction and RT-qPCR, and KR contributed to helpful discussions. All authors contributed to the article and approved the submitted version.

## Funding

FP, CF, CV, KP-B, AL, and KR were supported by NIH P20GM121288. DW was supported by NIH P20GM121288 and NIH P30GM114732. MMS was supported by U54 GM104940 from the National Institute of General Medical Sciences of the National Institutes of Health, which funds the Louisiana Clinical and Translational Science Center.

## Conflict of Interest

The authors declare that the research was conducted in the absence of any commercial or financial relationships that could be construed as a potential conflict of interest.

## Publisher’s Note

All claims expressed in this article are solely those of the authors and do not necessarily represent those of their affiliated organizations, or those of the publisher, the editors and the reviewers. Any product that may be evaluated in this article, or claim that may be made by its manufacturer, is not guaranteed or endorsed by the publisher.
