# Supplementary figures and images for: Attenuated Negative Feedback in Monocyte-Derived Macrophages From Persons Living With HIV: A Role for IKAROS
Source: Front Immunol. 2021 Nov 30;12:785905. doi: 10.3389/fimmu.2021.785905 (PMC8668949; doi:10.3389/fimmu.2021.785905)

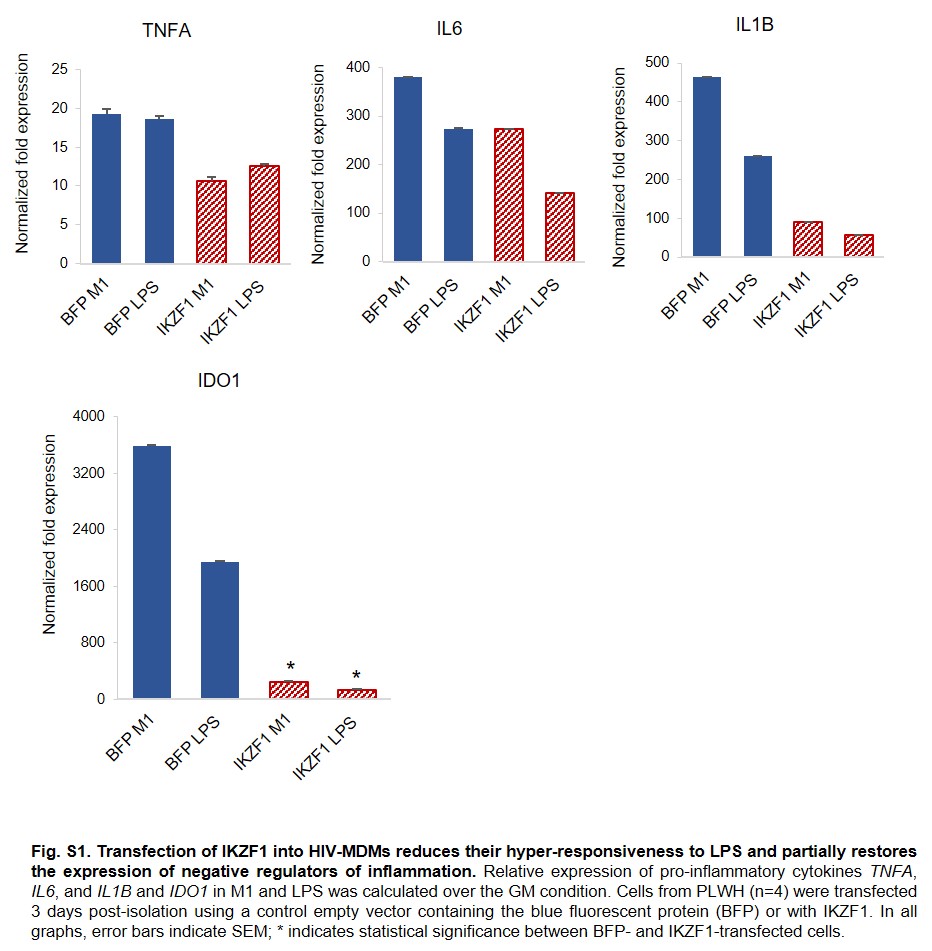

Supplement: Supplementary file 2 [file Image_1.jpeg]

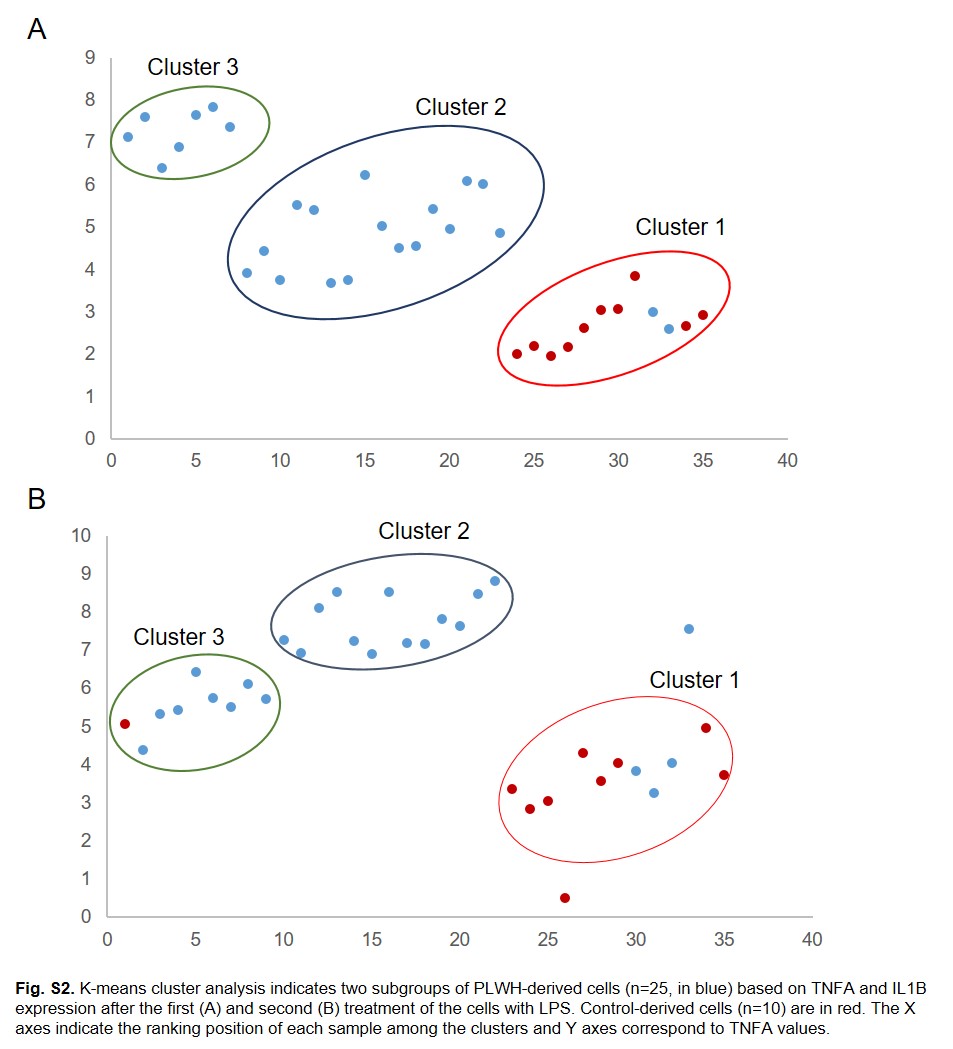

Supplement: Supplementary file 3 [file Image_2.jpeg]
