# Supplementary material for: Attenuated Negative Feedback in Monocyte-Derived Macrophages From Persons Living With HIV: A Role for IKAROS
Source: Front Immunol. 2021 Nov 30;12:785905. doi: 10.3389/fimmu.2021.785905 (PMC8668949; doi:10.3389/fimmu.2021.785905)
Supplement: Supplementary file 4 [file Table_1.docx]

Table S1. P-values corresponding to Fig. 5B (top) and 5C (bottom).

|  | TNFA | | IL6 | | IL1B | | NCOR2 | |
| --- | --- | --- | --- | --- | --- | --- | --- | --- |
|  | within | between | within | between | within | between | within | between |
| M1 | 1.9E-04 |  | 5.5E-04 |  | 1.0E-04 |  | 1.8E-02 |  |
| LPS | 1.4E-03 |  | 4.9E-03 |  | 2.1E-03 |  | 7.0E-01 |  |
| IKZF1 M1 | 1.2E-02 | 2.3E-05 | 8.3E-04 | 1.3E-03 | 7.3E-04 | 9.6E-03 | 1.9E-02 | 1.1E-03 |
| IKZF1 LPS | 2.5E-01 | 8.1E-02 | 9.1E-02 | 1.8E-01 | 4.1E-02 | 7.7E-02 | 2.2E-01 | 1.3E-01 |
|  | GSN | | HIVEP1 | | BIN1 | | IDO1 | |
|  | within | between | within | between | within | between | within | between |
| M1 | 5.9E-03 |  | 3.2E-03 |  | 5.2E-02 |  | 1.1E-04 |  |
| LPS | 3.3E-02 |  | 6.4E-02 |  | 2.9E-01 |  | 2.1E-03 |  |
| IKZF1 M1 | 8.3E-02 | 1.4E-02 | 9.2E-04 | 5.0E-02 | 6.8E-03 | 1.2E-01 | 5.8E-04 | 2.7E-05 |
| IKZF1 LPS | 2.2E-01 | 2.5E-01 | 9.5E-02 | 3.7E-01 | 3.6E-01 | 4.1E-01 | 1.4E-01 | 9.8E-03 |
