# Supplementary material for: Attenuated Negative Feedback in Monocyte-Derived Macrophages From Persons Living With HIV: A Role for IKAROS
Source: Front Immunol. 2021 Nov 30;12:785905. doi: 10.3389/fimmu.2021.785905 (PMC8668949; doi:10.3389/fimmu.2021.785905)
Supplement: Supplementary file 5 [file Table_2.docx]

Table S2. List of primers synthesized by IDT.

| Gene | Forward Primer 5’-3’ | Reverse Primer 5’-3’ |
| --- | --- | --- |
| *ABI1* | TCACTGTCACTGCCGACATC | AGTCTGGGGCTATTTGGCAC |
| *ACSL4* | TTTTGCGAGCTTTCCGAGTG | GGAAGCCGACAATAAAGTACGC |
| *ANKRD11* | TCCAGACACGGGATTTGTCG | TCAACCGTCTGCGATTCCAT |
| *BIN1* | CGTCAACACGTTCCAGAGCATC | CTTGACCGTGAAGGTGTTGCTC |
| *EGLN1* | GTTATCCGGGCAATGGAACG | GGGCTTTGCCTTCTGGAAAAA |
| *FBXW11* | GCAAGCTCTTAACTCTTCAGGTC | TGACACCAGGTTGCTGGCT |
| *GSN* | GGGACCAGGTCTTTGTCTGG | GGCTCAAAGCCTTGCTTCAC |
| *HIVEP1* | ACGAACCCATACAGATGTCCGC | CCTACTGAGACGCCTAAATCCAC |
| *IKZF1* | TCCCAAGTTTCAGGGAAGGAA | CTTGGAGCTTTGCTGTCCTCC |
| *IL1B* | TGGTCGGAGATTCGTAGCTG | TTCAAGCCCTTTCCTCCTCTC |
| *IL6* | AGACAGCCACTCACCTCTTCAG | TTCTGCCAGTGCCTCTTTGCTG |
| *ISG20* | ACACGTCCACTGACAGGCTGTT | ATCTTCCACCGAGCTGTGTCCA |
| *MSC* | GGTGGAGACACACTTCCCAG | GACAGACGCCTGCCTGATTA |
| *NCOR2* | CCACGTCATCTACGAAGGCAAG | CCTCCATCATGTCATAGGTGCG |
| *NFE2L2* | AGGTTGCCCACATTCCCAAA | AGTGACTGAAACGTAGCCGA |
| *OSBPL5* | AAGATCACGTCGGGAGAGGA | CAGAAAAGCGCACTGCTTCC |
| *OSM* | CATGGGGGTACTGCTCACA | CATGCTCGCCATGCTTGGAA |
| *PGAM5* | GTTCATGCCTCCCGACAAGA | AAACGTTAAGTGTGTGCGGC |
| *PPP1CB* | TATTTTCCGTGGGTGCCTCC | CTCGGCGTTCTCTCACCTAC |
| *PPP2R2B* | TATATCCCGGCTTGCTTGGG | GGTGCCCGAGGAATAACTGG |
| *PTGR2* | TGCCCGCAACACTGAGATAG | ATCATTGCTCTGGTTGGGCA |
| *PXMP2* | TGTGGACGCCACTACAGTTC | GTCGTCACTTCCCCAAGGAG |
| *RASA3* | GTCGACTGTGACATGCACCT | TCCCAATGTGATGAAGCCGA |
| *RB1* | CCTCCTTAATTTGGGAAGGTTTGT | GTGGCTTTGAACATGCCAGT |
| *RELA* | AGGCTATCAGTCAGCGCATC | TCCCCACGCTGCTCTTCTAT |
| *SIRPA* | CCCTCTACCTCGTCCGAATC | TGTGATATCATTTGTGTCCTGTGT |
| *SOCS1* | TCCCCTTCCAGATTTGACCG | GGGTACCCACATGGTTCCAG |
| *SOCS3* | GGGGAGTACCACCTGAGTCT | TGTGGTTGCTATCGTCCCAC |
| *SRGAP1* | GGACCCTGGGAGAAGGTCAT | GTGTTTCTGGGGCTTAGGGG |
| *SWAP70* | GATGAGGGTCCAGTGTCCAA | CCAACCTCCTCCCATAGCTTC |
| *TCF7L2* | ATCGTCCCAGAGTGATGTCG | ACTCAGCTACGACCTTTGCTC |
| *TNFA* | CTCTTCTGCCTGCTGCACTTTG | ATGGGCTACAGGCTTGTCACTC |
